# Supplementary material for: Comparing Control Intervention Scenarios for Raccoon Rabies in Southern Ontario between 2015 and 2025
Source: Viruses. 2023 Feb 14;15(2):528. doi: 10.3390/v15020528 (PMC9967127; doi:10.3390/v15020528)
Supplement: Supplementary file 1 [file viruses-15-00528-s001.zip › File S1.pdf]

File S1. Comparing SamPy to the Ontario Rabies Model

The Ontario Rabies Model (ORM) has been used and validated extensively for the purpose of modelling raccoon rabies in southern Ontario [7,10-12]. The transmission rate (β) used in ORM to model raccoon rabies in southern Ontario was 3.5% (i.e., the probability that the rabies virus is transmitted between an infectious raccoon and a noninfected raccoon during a contact; [7]). The actual transmission rate in nature is unknown. However, the rate of spread of rabies is commonly reported in field studies [12].

Since we were conducting raccoon rabies modelling in southern Ontario similar to that done by [7], we repeated the calibration analysis used by those authors as well as [12] to ensure SamPy predicts similar raccoon rabies spread rates to those predicted by the ORM and that a transmission rate of 3.5% should still be used. [12] analyzed the rate of spread of raccoon rabies by varying levels of K and the transmission probability (β) across a synthetic landscape to determine the sensitivity of the model to changes in β. Those rates of spread were then compared to rates of spread reported in the field in Ontario to determine the appropriate value of β. Values of β in southern Ontario and the northeastern United States were estimated to fall between 3 and 9%.

For our calibration analysis, we created a tube-shaped landscape of hexagonal cells (15 by 60 cells) similar to that used by [12]. We combined K values of 30, 60, 90, or 120 with β values of 3, 6, 9, or 12. For example, if a K value of 30 was used, all cells within the landscape were assigned a K value of 30. We conducted 20 trials for each of the 16 combinations. We used SamPy to build a raccoon population across each landscape over a period of 200 years. We seeded rabies in a random cell in the first column of the tube in year 200 and started the

simulations in year 201. We then replicated the calculation for the rate of spread of rabies based on columnar position that was used by [12] to determine if SamPy could replicate the results.

The results of the ORM simulations conducted by [12] when a K of 90 was used are directly comparable to Figure S1 which shows the results of our SamPy simulations when a K of 90 was used (please see File S1, page 7 of [7] for the ORM results). The predicted spread rates produced by SamPy were very similar to those predicted by ORM, suggesting SamPy was behaving similarly to the older, previously validated model. We also found, similar to the findings by [12], that simulations where K was 30 and β was 3 resulted in rabies dying out by column 3. The range of speeds and densities presented by [12], and replicated by SamPy, mimics what was seen in eastern Ontario, New York, and Vermont by the authors. In those areas, rabies did not spread in areas where animal densities were less than three animals/km^2 (i.e., a K < 30).

Since a transmission rate of 3.5% was used in these previous models of raccoon rabies for southern Ontario, where rates of rabies spread and the spatial distribution of K values would be similar, and we were able to replicate the relationship between spread rates and K values, we continued to use a β of 3.5%. However, it is important to note that this value was based on tests conducted on homogeneous landscapes and does not consider the control measures already in place in southern Ontario that will have affected the field measures of spread rate.

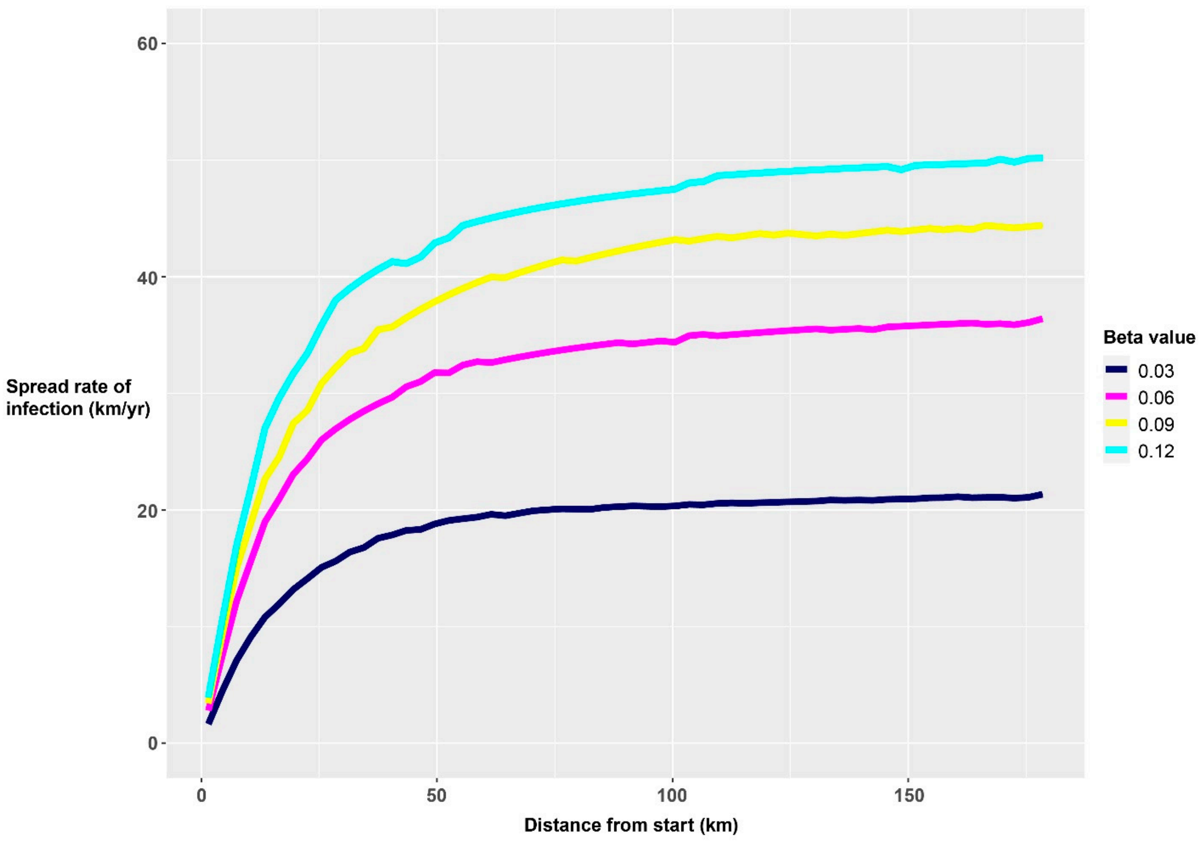

Figure S1. The rates of spread of raccoon rabies as predicted by SamPy are shown with transmission values (β, equivalent to 'sp') of 3, 6, 9, and 12 over a synthetic landscape where all cells have a K of 90.
