# Supplementary material for: Comparing Control Intervention Scenarios for Raccoon Rabies in Southern Ontario between 2015 and 2025
Source: Viruses. 2023 Feb 14;15(2):528. doi: 10.3390/v15020528 (PMC9967127; doi:10.3390/v15020528)
Supplement: Supplementary file 1 [file viruses-15-00528-s001.zip › File S2.pdf]

File S2: Calibration tests to determine the start week of rabies infections and vaccine levels

To determine both the week within which to seed rabies in the model landscape as well as the seroprevalence for each control intervention (i.e., the percentage of seropositive raccoons living in an area subjected to a disease control eight weeks following control intervention applications), we conducted a series of calibration tests. We tested the start week of initial rabies infections because, while the first case of raccoon rabies was detected in December of 2015, the actual timing of the first rabies infection(s) in the raccoon population remains unknown.

We tested seroprevalence because observed seroprevalence rates do not align with observed outbreak containment results and, in some areas, showed relatively large variations between years (MNRF, Peterborough, Ontario, Canada, 2022). For instance, annual estimates of seroprevalences in raccoon populations sampled in urban areas varied from 6 to 18%, while those in rural areas varied from 16 to 37% between 2016 and 2019. In addition, the serology tests were based on a titre of 0.5 international units per ml (IU/mL) [16]. This titre differs from that used in the United States, where titres can be as low as 0.125 IU/mL [24]. The actual percentage of raccoons with protective rabies titres resulting from rabies control interventions remains imprecise.

For each calibration test, we maintained all input parameters as those specified in Table 1 and varied both the start week and the seroprevalence. We varied the start week between Week 40 and Week 48 of 2015 (where Week 48 coincided with the first week of December 2015, the week the first raccoon rabies case was detected) [6]. For seroprevalence, we multiplied the measured seroprevalences for each control intervention type as specified by the MNRF (MNRF, 2021) by a factor of between 1 and 3 at increments of 0.2. Trap-vaccinate-release (TVR) strategies have increased certainty in seroprevalence due to animal marking and direct

intramuscular vaccination of raccoons by control officials. We therefore left TVR levels at their measured seroprevalence of 60% across all tests.

The combinations of start weeks and seroprevalence variations resulted in 99 calibration tests. For each calibration test, we conducted 100 trials and calculated the median number of new raccoon rabies infections in raccoons per month along with the upper and lower quartiles. We then compared the predicted distribution of monthly infections to the actual numbers detected by the MNRF between December 2015 and September 2021 (Figure S2.1). For each detected raccoon case recorded by the MNRF, at least two dates were reported. The date collected was the date when the animal was euthanized or found dead, while a mapping date was the date the positive was actually reported as part of the surveillance program. For our modelling purposes, we used the earliest date available per case.

If two consecutive increments produced similar epicurves, we conducted the tests for the 0.1 increment in between (e.g., if tests where seroprevalences were multiplied by 2.6 and 2.8 produced similar epicurves that closely resembled the actual epicurve, we tested seroprevalences multiplied by 2.7 across all weeks). Since the actual numbers were detected in and around the Hamilton area, we narrowed our counts of predicted infections to a similar region, using only grid cells that fell within 27 km of confirmed raccoon rabies cases (n = 1,592 grid cells). We used 27 km because this is the maximum dispersal distance used in our model (i.e., raccoon agents can travel a maximum of eight cells, equivalent to ~27 km, per year).

To compare each predicted distribution of monthly infections to the actual monthly cases detected (Figure S2.1), we converted both the actual and predicted distributions of infections to percentages of the total number of infections between December 2015 and September 2021 and performed both a Kolmogorov-Smirnov (KS) test as well as a simple linear regression analysis.

For the KS tests, seroprevalences multiplied by 2.1 with simulations starting in weeks 46 and 47 tied for showing the least disimilarities (i.e., the smallest standardized mean difference between the two distributions) (D = 0.173, p = 0.178). For the linear regression analysis, where the actual distributions were a function of the predicted distributions, seroprevalences multiplied by 2.1 with simulations starting in week 47 produced the highest R^2 value ($R^2 = 0.597$, $p < 10^{-6}$). We therefore ran our models using seroprevalences multiplied by 2.1 and seeding rabies in week 47 (the third week of November) of 2015 (Figure S2.1).

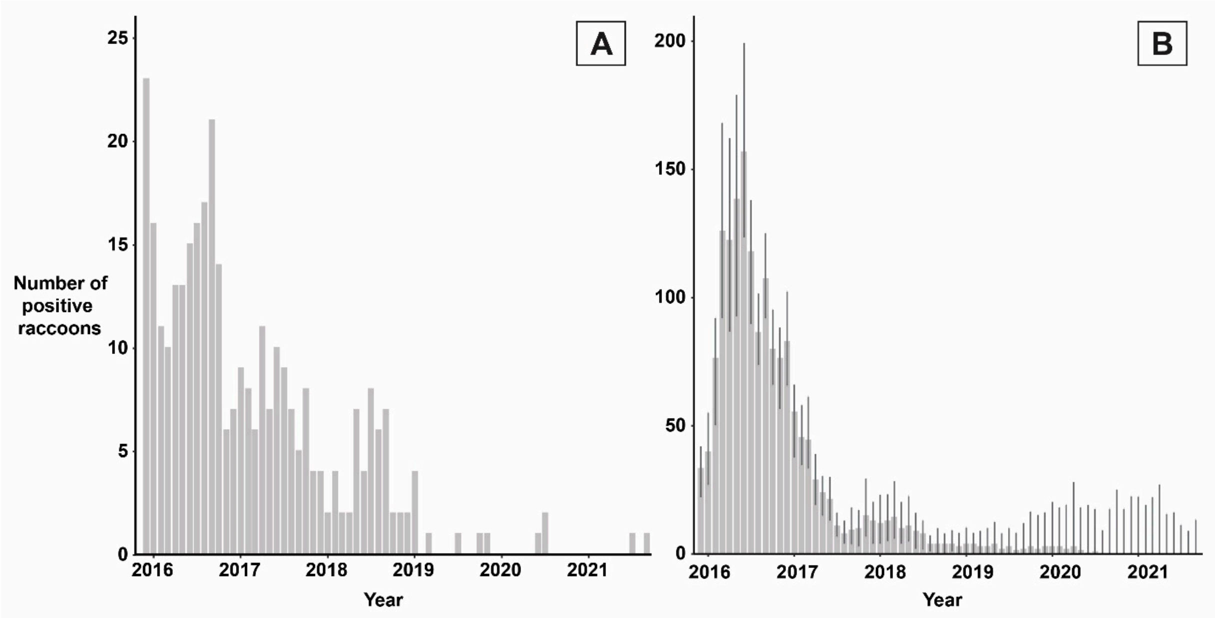

Figure S2.1. Epicurves at a monthly time scale for A) detected cases of raccoon rabies by the Ministry of Natural Resources and Forestry and B) predicted median cases of raccoon rabies with seroprevalences multiplied by 2.1 and a start week of 47 (n=100). Detected case numbers are based on the earliest available date the animal was euthanized or found dead and not based on when the positive animal was reported as part of MNRF surveillance.For the epicurve of predicted cases, the upper and lower quartiles are also shown.
