# Supplementary material for: Comparing Control Intervention Scenarios for Raccoon Rabies in Southern Ontario between 2015 and 2025
Source: Viruses. 2023 Feb 14;15(2):528. doi: 10.3390/v15020528 (PMC9967127; doi:10.3390/v15020528)
Supplement: Supplementary file 1 [file viruses-15-00528-s001.zip › File S3.pdf]

File S3. Vaccine application and resulting immunity in modelled raccoons in SamPy

In this project, seroprevalences for each control intervention type were provided by the Ministry of Natural Resources and Forestry and represented the percentage of seropositive raccoons living in an area subjected to a disease control eight weeks following control intervention applications. We tested how SamPy predicted immunity in raccoons when applying controls in one year, or every year, over a five-year period across a synthetic landscape. To do this, we used the same synthetic tube-shaped landscape as that used in File S1 (15 by 60 cells; [10]) and applied a K of 90 in all cells to ensure the raccoon population was not at risk of dying off. We tested seroprevalences of 11, 20, 24, and 60, which were the four seroprevalences used in our study for hand baiting, bait stations, fixed wing/helicopter, and trap-vaccinate-release interventions, respectively. We applied each seroprevalence in Week 34 of 2015 (one-year tests), or week 34 of each year from 2015 through 2019 (five-year tests). Each combination of seroprevalences and application frequencies was run 100 times, resulting in 800 trials.

For each combination of seroprevalences and application frequencies, we extracted the number of immune raccoons per cell every week starting four weeks before through 208 weeks after application of the control. For each week, we calculated the total number of immune raccoons and the total population of raccoons across all cells and all simulations. We then divided the total number of immune raccoons by the total population of raccoons to determine the percentage of immune raccoons per week across the landscape.

For the one-year tests, each seroprevalence showed the same increase in immunity over time, with the percentage of immune raccoons increasing from 0% to the percent of the seroprevalence by week 35 (one week after the week of vaccine application, Figure S3.1). By week 70 (i.e., 36 weeks after the week of application, which is also week 18 of the next year), the

population experiences a birth pulse, causing a drop in the percentage of immune raccoons. Following the birth pulse, immunity increases as raccoons naturally die over the year. In the next two years, two more birth pulses occur, causing immunity to decrease before dropping to 0% by week 190 (i.e., 156 weeks, or three years, after the week of application). This decrease to 0% at week 190 is due to immunity lasting in the modelled raccoons for three years.

We assumed a three-year period of protection in raccoons with vaccination based on the licensed use of Intra Muscular (IM) rabies vaccination in dogs, cats, and sheep with IMRAB vaccine [25] which was used in the TVR vaccinated animals in southern Ontario. Available data in raccoons reported 10/11 raccoons vaccinated IM with IMRAB had protective titres when measured 300 to 757 days post vaccination [26]. [27] challenged raccoons with rabies virus that had been vaccinated with an IM IMRAB vaccine 442 days prior. 13/18 of the raccoons survived (all of the raccoons that showed an initial serological response survived). While the available data from longitudinal Ontario Rabies Vaccine Baits (ONRAB) oral vaccination challenge studies are more limited, studies from [28] and [29] have so far reported 75% and 85% of raccoons had effective protection from rabies challenge at 180 days and 350 days post vaccination, respectively.

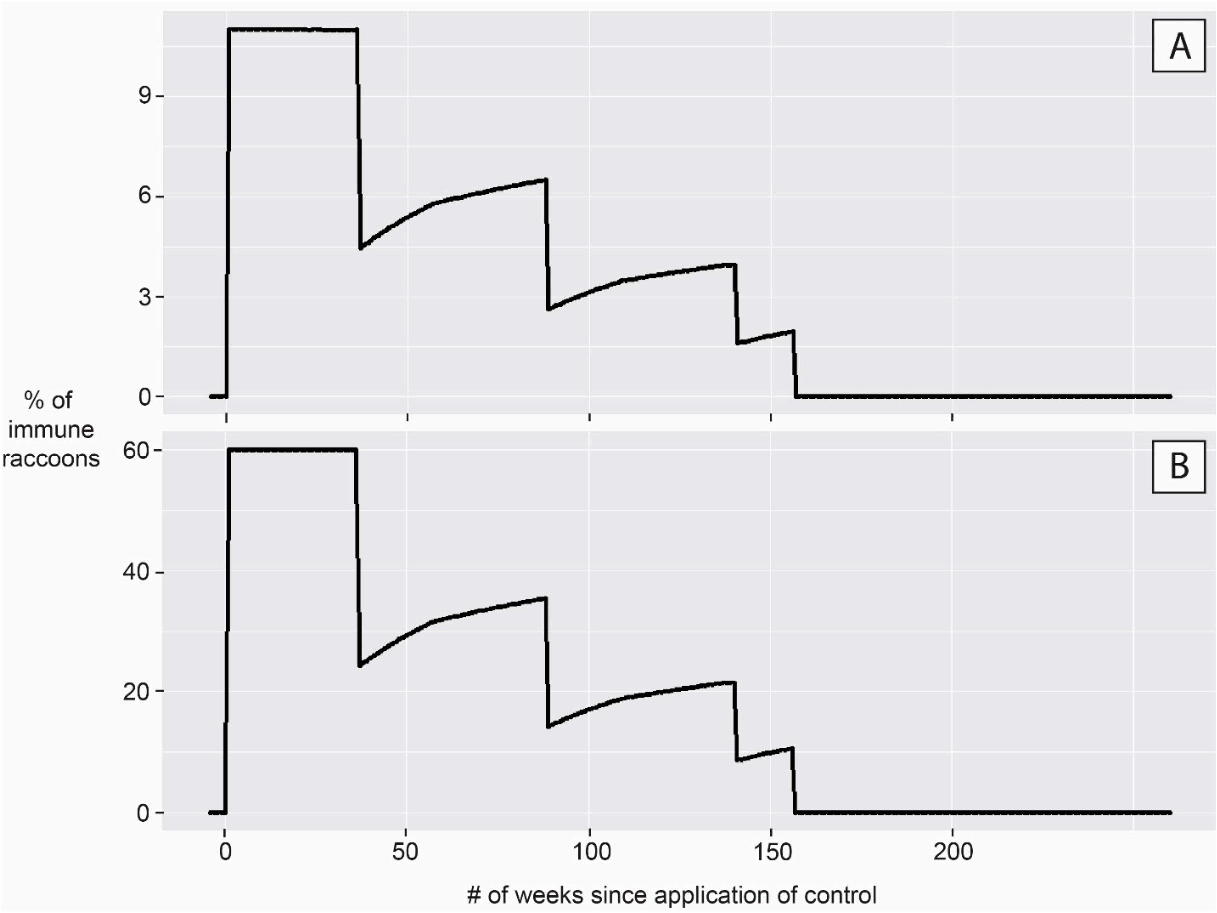

Figure S3.1. The percentage of immune raccoons per week for five years when a control with a seroprevalence of A) 11% and B) 60% is applied once in week 34 of the first year. Percentages are shown each week starting four weeks prior to, until 208 weeks after, the application of the control.

For the five-year tests, each seroprevalence also showed the same increases and decreases in immunity over time with the percentage of immune raccoons increasing from 0% to the percent of the seroprevalence by week 35 of the first year of vaccine application, followed by a drop in immunity during the birth pulse of the following year (Figure S3.2). However, in contrast

to the one-year tests, every time vaccines were applied (once per year, at week 34 each year), seroprevalence increased again by the seroprevalence value in the second year, but increased by less than the seroprevalence value in the subsequent years. For example, when applying a seroprevalence of 11% every year for five years, immunity increased 11% the week after vaccine application for the first two years. In the third year, immunity increased ~10% the week after vaccine application. After the third year, when immunity in vaccinated raccoons began to expire and raccoons that received a vaccine in the first year reach the end of their lifespan, immunity leveled off around 18-20% (Figure S3.2A). As the seroprevalence of a control increased, immunity following vaccine application would increase by smaller percentages in subsequent years. This is because a higher percentage of raccoons each year would have already received a vaccine, only to receive one again. For example, when applying a seroprevalence of 60% every year for five years, immunity increased 60% the week after vaccine application for the first year, then increased ~42% the following year. In the third year, immunity increased to nearly 80% and then plateaus as immunity in vaccinated raccoons began to expire and raccoons reached the end of their lifespan (Figure S3.2B).

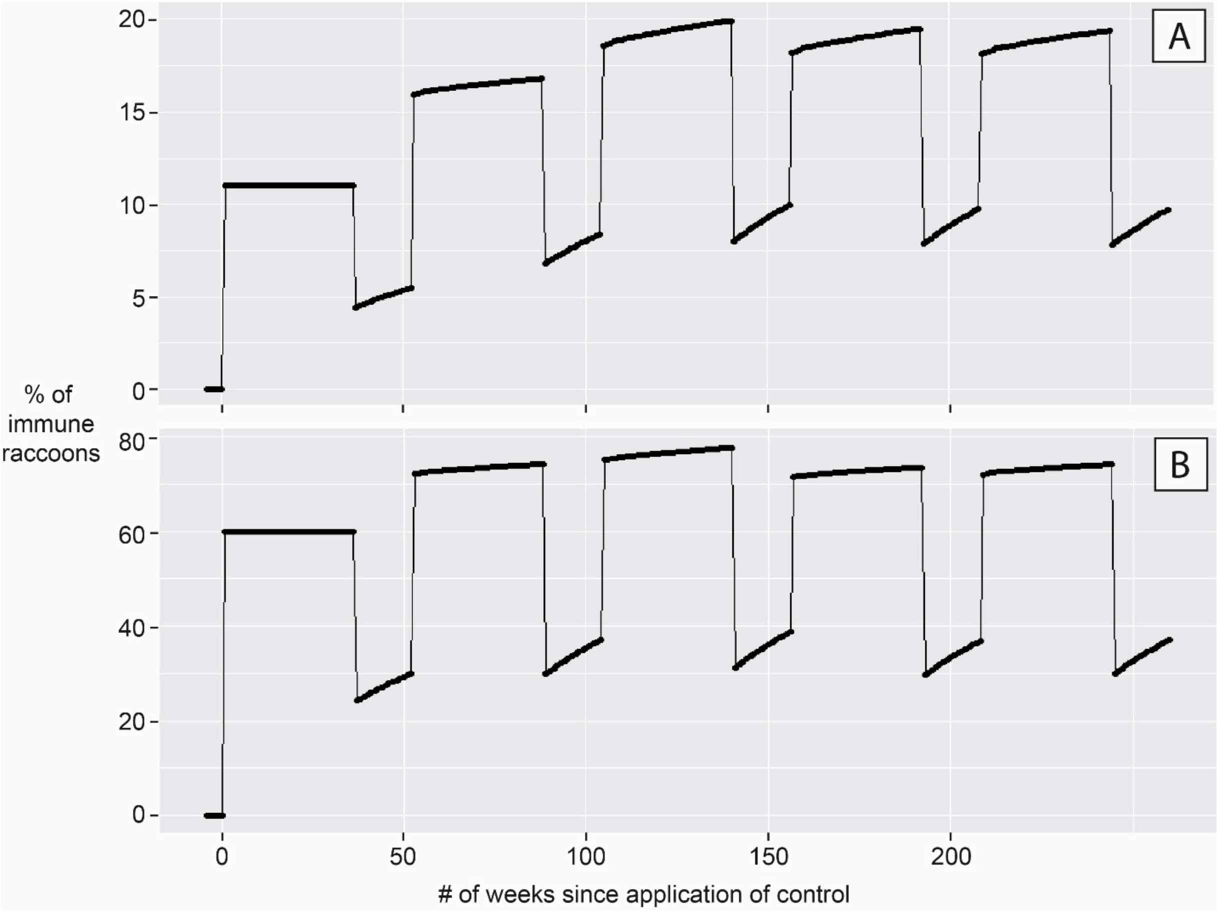

Figure S3.2. The percentage of immune raccoons per week for five years when a control with a seroprevalence of A) 11% and B) 60% is applied once in week 34 of every year for five years. Percentages are shown each week starting four weeks prior to, until 208 weeks after, the application of the control.

Across all tests, the level of immunity appears relatively stable from Week 34 of 2015 until the birth pulse in Week 18 of 2016. This initial plateau in immunity is caused by all raccoons being uniformly vaccinated across all age classes without the introduction at any point of new, unvaccinated raccoons due to new births. When the birth pulse in Week 18 of 2016 occurs, a new population of unvaccinated newborn raccoons enters the population, causing

overall immunity of the population to decrease by Week 19. Raccoons in their first year of life, however, have higher rates of mortality than raccoons in their second, third, or fourth year of life. Therefore, the level of immunity of the overall population gradually increases until the next birth pulse due to die-offs of young, unvaccinated raccoons.
