# Supplementary material for: Comparing Control Intervention Scenarios for Raccoon Rabies in Southern Ontario between 2015 and 2025
Source: Viruses. 2023 Feb 14;15(2):528. doi: 10.3390/v15020528 (PMC9967127; doi:10.3390/v15020528)
Supplement: Supplementary file 1 [file viruses-15-00528-s001.zip › Table S1.pdf]

Table S1. Raccoon density index (RDI) and capacity of support (K) based on field studies and estimates based on expert opinion (E.O.; T. Buchanan and L. Nituch). K values were calculated by multiplying the RDI value for each land variable by 10.

**Land variable**	**RDI (number of raccoons per km²)**	**Capacity of support (K) used in model landscape**	**Source**
Single variable grid cells			
Coniferous forest	2.9	29	[18]
Deciduous forest	3.4	34	[20]
Elevation above 500 m	1.0	10	[18]
Exposed land / Barren	3.4	34	[20] and E.O.
Grassland	3.4	34	[20] and E.O.
Mixed forest	3.2	32	Average of densities reported for coniferous and deciduous forests as reported by [18] and [20]
Pasture	5.1	51	[19] and E.O.
Shrubland	3.4	34	[20] and E.O.
Urban	18	180	[18]
Peri-urban	19	190	[18]
Water	0	0	[18]
Wetland	3.4	34	[20] and E.O.
Cultivated cropland: Corn	13.6	136	[20] and E.O.
Other cultivated cropland<br>■ Agriculture (undifferentiated)<br>■ Barley<br>■ Greenhouses<br>■ Oats<br>■ Orchards<br>■ Other vegetables<br>■ Rye<br>■ Soybeans<br>■ Tobacco<br>■ Vineyards<br>■ Wheat	12.5	125	[19] and E.O.
Variable combinations per grid cell			
Corn + deciduous + peri-urban	30	300	[19] and E.O.

Corn + deciduous + peri-urban + urban	30	300	[19] and E.O.
Corn + deciduous + urban	27	270	[19] and E.O,
Deciduous + peri-urban	25	250	[19] and E.O.
Deciduous + peri-urban + urban	25	250	[19] and E.O.
Deciduous + urban	21	210	[19] and E.O.
Corn + deciduous	18	180	[20] and E.O.
